# Flow Structure Investigations during Novec Refrigerant Condensation in Minichannels

**DOI:** 10.3390/ma14226889

**Published:** 2021-11-15

**Authors:** Małgorzata Sikora

**Affiliations:** Department of Energy, Koszalin University of Technology, ul. Racławicka 15-17, 75-620 Koszalin, Poland; malgorzata.sikora@tu.koszalin.pl

**Keywords:** flow structures, condensation, minichannels, flow maps, image analysis

## Abstract

This article presents the results of flow visualization studies of Novec refrigerants during condensation in minichannels. Experimental investigation of two-phase flow regimes was conducted in minichannels with internal diameter *d_h_* = 2.5, 2.0, 1.2, and 0.5 mm. Images of the two-phase flow structures were analyzed by using the MATLAB algorithm. To calculate void fraction, a two-dimensional areal quantitative stereology technique was used. Observation of flow structures formed during the process of condensation was the major aim of the investigations. The condensation studies were conducted over a wide range of mass flux densities (G = 80–5500 kg/m^2^s) and saturation temperatures (*t_s_* = 30–70 °C). Visualization results and image analysis methods are described in this paper. Based on the experimental results, a flow structure map was constructed and presented.

## 1. Introduction

The issues of heat and mass flow are of great importance in current technological development [1,2]. Two-phase flows with heat exchange play an important role here. The flow structures formed during the condensation process have a significant impact on the intensity of the condensation process. It is influenced by void fraction *φ*, vapor quality *x*, the mass flux density *G*, and the shape of the interface. For this reason, in addition to the study of heat transfer or flow resistance during the condensation process in minichannels, the observation of two-phase flow structures formed in a nonadiabatic flow is of great importance. In a nonadiabatic flow, it is very important to define the range of the above-mentioned parameters in which individual flow structures occur. The structures of two-phase adiabatic flow; e.g., water and air, are the most widely described in the literature. However, in heating and refrigeration devices, two-phase nonadiabatic flows, such as boiling or condensation, are most often used. Due to the difficulty level of the condensation process and its observation in minichannels, studies of the flow structures in the boiling process are conducted and described much more frequently. The investigations of Elazhary and Soliman [3] are an example of research on adiabatic flow structures. The authors conducted experimental investigations of two-phase flow regimes and pressure drop in horizontal minichannels with a rectangular cross-section and dimensions of 1.87 × 20 mm^2^. They investigated the flow of air–water mixtures at 200 kPa pressure and standard temperature. The authors identified bubbly, plug, churn, and annular flow structures. The studies were conducted in gas and liquid phases with superficial velocities ranging from 0.04 ≤ *j_v_* ≤ 10 m/s to 0.02 ≤ *j_l_* ≤ 0.7 m/s. The most extensive studies regarding condensation processes in minichannels were carried out by Coleman and Garimella [4]. They investigated the condensation of R134a refrigerant in nine minichannels of various cross-sections (round, square, and rectangular) with a hydraulic diameter *d_h_* = 1–4.91 mm and mass flux density in the range *G* = 150–50 kg/m^2^s. The channels were cooled by air. The flow was visualized during the condensation process, and based on these findings, the flow structures and substructures were classified. Four structures were predominantly observed: annular, wave, intermittent, and dispersed. The influence of gravity, interfacial interactions, and inertial force was also studied. It has been shown that the changes in vapor quality and mass flow influence the flow structure. The impact of the channel size on the existing structures has also been demonstrated [5]. On the other hand, Jige et al. [6] investigated the condensation of R32 refrigerant in rectangular multiports with a hydraulic diameter *d_h_* = 0.5–1 mm. The results demonstrated that the plug structure occurred at low vapor quality, while the annular structure occurred at high vapor quality and high mass flux density. Xiao and Hrnjak [7] conducted research on the condensation of R134a, R1234ze, R32, R245fa, and R1233zd refrigerants in channels with a hydraulic diameter *d_h_* = 1.4–6 mm. The transition between various structures formed during the process of condensation was described. The annular flow is mainly characterized by the presence of surface tension and shear forces. As the thickness of the condensate film increases, the gravity force obtains a value higher than the surface tension and shear forces. At this point, the flow changes to stratified flow. When the thickness of the liquid film approaches the radius of the channel, the ratio of gravity force to the surface tension can be given as the Bond number Bo:

(1) $$Bo=\frac{\left(\rho_{l}-\rho_{v}\right)\cdot g\cdot d_{h}^{2}}{\sigma },$$

where *ρ_v_* and *ρ_l_* are the densities of the gas and liquid phases, respectively; *d_h_* is the hydraulic diameter; *g* is the gravity acceleration; and *σ* is the surface tension factor. The relationship between inertia forces and surface tension can be described by the Weber number (*We*):

(2) $$We=\frac{G^{2}\cdot d_{h}}{\sigma \cdot \rho_{v}}$$

The Weber number is used for calculations in two-phase flows, with particular emphasis on disturbances at the interface. This number describes the ratio of inertia forces to surface tension. The transition between stratified and wave structures is caused by the formation of Kelvin–Helmholtz instability, and the height of the waves depends on the differences in the velocity of the liquid and gas phases. For a transition to an intermittent flow, the wave amplitude should be close to the internal diameter of the channel (or otherwise should reach the highest value). At low values of the mass flux density *G*, the amplitude of the waves is low, which prevents the formation of an intermittent flow despite an increase in the thickness of the liquid film. Enoki et al. [8] observed the flow structures of two-phase R410A refrigerant in circular, rectangular, and triangular cross-sectional channels with a hydraulic diameter *d_h_* = 1 mm. The study was carried out in the field with a mass flux density *G* = 30–400 kg/m^2^s, vapor quality *x* = 0.05–0.9, and saturation temperature *t_s_* = 10 °C. The results revealed the formation of slug, stratified, wave, froth, and annular flow structures. On the basis of these results, the authors made modifications to the flow structure map developed by Chen et al. [9], which was created on the basis of a two-phase flow test of the R134a refrigerant in pipe channels with hydraulic diameter *d_h_* = 1.10, 2.01, 2.88, and 4.26 mm [6]. Nema et al. [10] proposed new transition boundaries for individual flow structures, using the database for the condensation of R134a refrigerant in the channels with a hydraulic diameter *d_h_* = 1–4.91 mm and mass flow density range *G* = 150–750 kg/(m^2^s). Thus, they proved the influence of the channel diameter and heat transfer on the occurrence of some flow structures, by comparing it with adiabatic, air–water, two-phase flow under similar conditions. In the case of adiabatic flow, the occurrence of “discrete wave” and “dispersed wave” structures was not observed, and the boundaries of individual structures differ significantly in both cases. It is similar to the influence of the channel diameter on the range of occurrence of particular structures. The decrease in the diameter of the channel causes the shifting of most of the boundaries towards a higher vapor quality *x*. This shift is in turn affected by an increase in the influence of viscosity *μ* and surface tension *σ* on the condensation process, following a decrease in the diameter of the channel and the influence of gravitational forces. Nema et al. [11] used the Bond number (*Bo*) to determine the formation of the wave structure during the flow. When the Bond number exceeds the critical value (*Bo_cr_*), there is a possibility for the occurrence of a wave structure. Otherwise, there is a direct transition from the annular to an intermittent structure. The critical Bond number is determined from the following equation:

(3) $$Bo_{cr}=\frac{1}{\left(\frac{\rho_{l}}{\rho_{l}-\rho_{v}}-\frac{\pi }{4}\right)}$$

If the Bo number is lower than *Bo_cr_*, the minimum amount of liquid phase required for the formation of gas slugs is described by the Lockhart–Martinelli parameter *χ_tt,slug_*:

(4) $$\;\;\;\;\;\;\;\;\;\;\;\;\;\chi_{tt,slug}=\chi_{tt,0}=0.3521\;dla\;Bo\leq Bo_{cr},$$

(5) $$\;\;\;\;\;\;\;\;\;\;\;\;\chi_{tt,slug}=\chi_{tt,0}+\frac{\chi_{tt,1}\left(Bo-Bo_{cr}\right)}{Bo-Bo_{cr}+5,5}\;dla\;Bo>Bo_{cr}$$

The transition between a discrete wave and dispersed wave structure is described by the modified Froude number Fr*, which determines the mutual relationship between inertia force and gravity in the flow:

(6) $$Fr^{*}=\frac{G\cdot x}{\sqrt{d_{h}\cdot g\cdot \rho_{v}\cdot \left(\rho_{l}-\rho_{v}\right)}}.\;$$

Zhuang et al. [12] performed a study on the two-phase flow of R170 refrigerant in a horizontal, smooth glass channel with a hydraulic diameter *d_h_* = 4 mm. During the investigation, structures such as slug, transitional, annular wave, and annular were observed. The experimental results were compared with the maps constructed by Breber, Tandon, Cavallini, El Hajal, and Barbieri, and then the authors modified the map developed by Kim et al. [13]. They also determined the limits of transition between individual flow structures using the modified Weber number (We*) and the Lockhart–Martinelli parameter:

- Annular/annular wave: $We^{*}=29.25\chi_{tt}^{0.27};$
- Annular wave/transitional: $We^{*}=18.91\chi_{tt}^{0.33}$;
- Transitional/slug: $We^{*}=9.62\chi_{tt}^{0.35}$;
- Slug/plug: $We^{*}=4.38\chi_{tt}^{0.45}$.

In the process of identifying two-phase flow structures, flow structure maps are a very useful tool. Nasrfard et al. [14] analyzed the efficacy of flow maps proposed by El Hajal et al., Kim et al., Tandon et al., and Cavalini et al. by comparing them with the results of their own experimental studies on the condensation of R141b refrigerant in a horizontal, smooth circular channel with a hydraulic diameter *d_h_* = 8 mm. It was found that the previously described maps of flow structures did not match with the results of visualization. Therefore, the authors proposed their own map, which was a modification of the El Hajal et al. map that functions in the low range of the vapor quality values. Xiao and Hrnjak [7] proposed a map of the flow structures formed during the condensation of R134a, R1234ze, R32, R245fa, and R1233zd refrigerants in the channels with hydraulic diameter *d_h_* = 1.4 and 6 mm. Specific enthalpy of the refrigerant was adopted as one of the parameters that define the limits of the occurrence of individual flow structures. This procedure took into account the heat transfer that occurred during the process of condensation. The map thus constructed was also a modification of El Hajal et al.’s map.

In the literature, there are many adiabatic two-phase flow studies. Some authors published a flow structures map for adiabatic and nonadiabatic two-phase flows. There is a lack of visualization studies of condensation of new, environmentally friendly refrigerants with low ODP (Ozone Depletion Potential) and GWP (Global Warming Potential). The condensation process of these refrigerants in minichannels has not been tested in a wide range. Flow structure maps for the condensation process of new refrigerants are lacking in the literature. The novelty of this work is represented by the investigation results of the condensation flow structures of new low-pressure refrigerants from 3M’s Novec group in minichannels. The study was conducted over a very wide range of condensation processes in minichannels. So far, only Al-Zaidi et al. [15] investigated the flow structures formed during the condensation of HFE7100 refrigerant in a multiport made of rectangular minichannels with *d_h_* = 0.57 mm, *t_s_* = 60 °C, and *G* = 48–126 kg/m^2^s. Mikielewicz et al. [16] carried out investigations on HFE 7000 and HFE 7100 refrigerant condensation in a minichannel with an internal diameter *d_h_* = 2.3 mm for the mass flux density range *G* = 240–850 kg/m^2^s, but the authors did not investigate the flow structures during condensation of these refrigerants. The Novec refrigerants are used in wide range of heating devices. Woodoc et al. [11] investigated the use of HFE7000 refrigerant in a silicone mini heat exchanger (MECH-X), which is a heat sink reactor with a thickness of 800 μm. The exchanger was used to cool electronic components that were heated to 90 °C. The boiling studies of Novec refrigerants (HFE7000, HFE7100, FC-72, and HFE-649) were conducted by Eraghubia et al. [17], Piasecka et al. [18,19,20], and Cao et al. [21], who created a visualization of pool boiling of HFE-649. Mohamadi et al. [22], on the other hand, investigated heat transfer with the use of a nanofluid that consisted of an HFE 7000 refrigerant and Al_2_O_3_ as well as SiO_2_ nanoparticles. Adebayo et al. [23] proposed the use of an HFE 7000 refrigerant in combination with CO_2_ in a cascade refrigeration device. Other scientists proposed the use of Novec refrigerant in the organic Rankine cycle [24] and a three-stage refrigeration system [25]. Currently, two-phase flow modeling also is very often used to reduce costly experimental studies in the field of flow structures [26].

The next novelty of this article is represented by flow structure maps, developed based on visualization and flow investigations. So far, no results of flow structures during the process of condensation of this type refrigerants in minichannels have been published. The proposed flow structure maps are also new in this field.

## 2. Test Section

**Figure 1:**
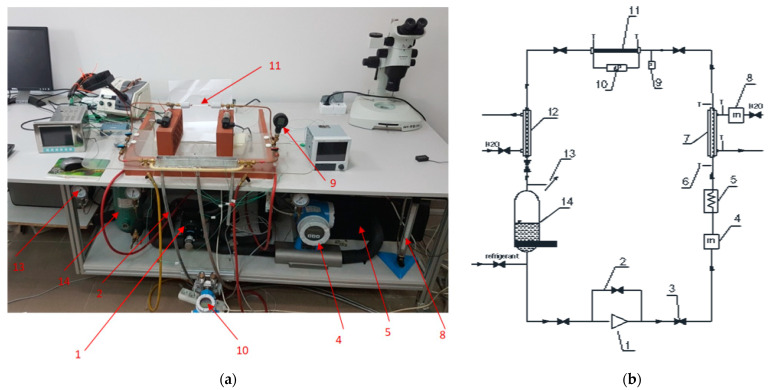
Test stand: (**a**) overview; (**b**) schematic diagram; 1—ceramic pump, 2—bypass for flow rate control, 3—valve, 4—electronic flow meter for refrigerant, 5—evaporator, 6—manometer, 7—preliminary heat exchanger of “tube-in-pipe” type, 8—flow meter for water flow rate measurement, 9—refrigerant pressure sensor on inlet to measuring section, 10—refrigerant pressure difference meter, 11—horizontal section for flow visualization, 12—heat exchanger (subcooler), 13—pressure gauge, 14—liquid refrigerant tank.

**Figure 2:**
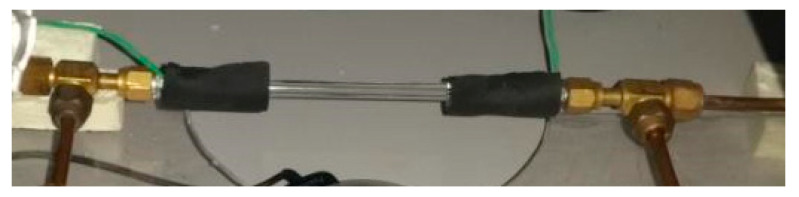
Overview of the measuring section 12.

Condensation and visualization studies of HFE7000, HFE7100, and Novec649 refrigerants were performed on a specially designed and built test stand (Figure 1). The measuring section marked as 12 in Figure 1 is shown in Figure 2. It consisted of a minichannel made of glass, which was used for the visualization of the two-phase flow of refrigerant during the condensation process. For the experimental purpose, the refrigerant liquid was drawn by a ceramic pump 1 and forced into a heat exchanger 5, which consisted of a pipe coil immersed in a water tank (it acted as an evaporator). Through a system of electric heaters, heat flux was applied to the water bath in which the pipe coil with refrigerant was immersed. The refrigerant was heated until it evaporated. The temperature of the steam leaving the evaporator was measured and kept at a constant level by using a thermostat. A Coriolis 34XIP67 mass flow meter 15 with an accuracy class of 0.5 was installed at the inlet where the refrigerant entered the heat exchanger. At the inlet of the measuring section 12, a precooled heat exchanger was installed to adjust the vapor quality 8. Based on the energy balance in the heat exchanger, the value of the vapor quality x at the inlet to the measuring section was determined. Temperature sensors were installed at the inlet and outlet of the minichannel (with a thermocouple diameter of 0.1 mm). The pressure of the refrigerant in the measuring section was measured using a piezoresistive sensor 10 fitted with a PMP131-A1401A1W transducer (manufactured by Endress + Hauser, Reinach, Switzerland) with a measuring range of 0–40 MPa and a class of 0.5. The pressure drop in the minichannel was measured using a differential pressure sensor (Endress + Hauser, Maulburg, Germany) fitted with a Deltabar SPMP transducer 11 with a measuring range of 0–1.5 MPa and a class of 0.075. At the outlet of the measuring section, a subcooler 14 was placed. Then, the refrigerant liquid was directed to the liquid tank. A bypass system 2 with valves was also installed to regulate the flow rate of the refrigerant. In the measuring section, an Olympus i-SPEED 3 camera (Olympus, Shanghai, China) with a CMOS sensor (with a maximum recording speed of 10,000 fps and a maximum resolution of 1280 × 1024 pixels) and a software and data acquisition system was installed. For experimental purposes, an AF-S VR Micro-Nikkor 105 mm f/2.8 G IF-ED lens and a Nikon AF 18–35 mm f/3.5–4.5 D IF-ED wide-angle lens were used.

**Figure 3:**
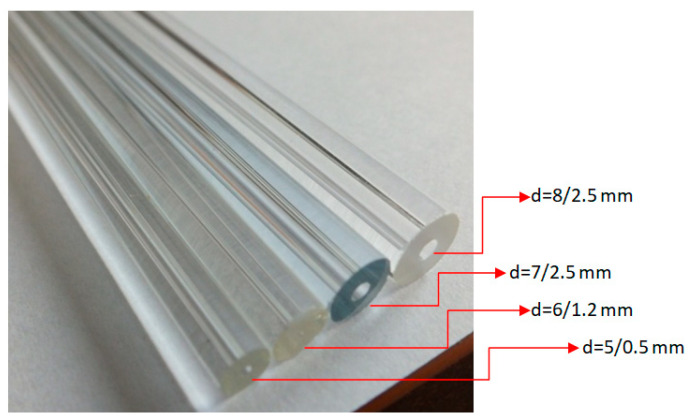
Overview of the used glass minichannels.

The visualization tests were performed in the glass minichannels (Figure 3). The channels with diameter *d_h_* = 2 mm, 1.2 mm, 0.8 mm, and 0.5 mm and a length of 250 mm were selected for the studies [27,28,29]. The flow structures formed during the condensation of HFE7000, HFE7100, and Novec649 refrigerants in the minichannels were visualized by recording videos using a slow-motion camera. The camera recorded the images of the flow on the “side” of the channel so that it was possible to enter the path where the gravity influenced the flow structure formed. The frequency of recorded images and the power of lighting were regulated depending on the flow velocity in the minichannel. The higher flow velocity required a number of captured images. The increase in the number of recorded frames per second caused the “illumination” of the measuring section, indicating that the lighting power had to be increased. When the visualization studies of flow structures were conducted in the glass minichannels with a circular cross-section, it was important to avoid refractions and reflections of the light on the surface of the channel. The angle of light incidence on the channel and the degree of its dispersion influenced this phenomenon.

## 3. Image Analysis

The term “image analysis” refers to the automatic processing and analysis of images of selected objects or surroundings, and the primary objective is to obtain useful mathematical information that can be used for their interpretation that may further affect the results of the observed process. The image analysis algorithms process the images in three stages. The first stage involves preprocessing of the video signal. Its purpose is to eliminate disturbances, extract the examined object from the background, determine the level of disturbances, and balance the histogram. The second stage includes image segmentation, location of the examined objects, recognition of their shape, distinguishing the characteristic features of the object, etc. The final stage comprises analysis of the movement of the examined object, if any; interpretation of the object control; assigning processing parameters; and analyzing the image [30].

Visualization studies of adiabatic, two-phase, gas–liquid flow in vertical channels have been carried out by many authors [31,32]. Quantitative stereology technique was used in this work. Stereology is a scientific discipline in which information about the three-dimensional structure of objects can be obtained from their two-dimensional counterparts (mainly images). It provides information about volume (*V*), surface area of the object (*A*), and length of the line (*L*). The application of stereology methods requires the inclusion of probability calculations and mathematical statistics because the studied structures are sometimes stochastic, as their sizes and shapes change over time and space (especially during boiling and condensation processes). In stereology, one dimension is reduced at the moment it is mapped on the solid plane. Reduced quantities indicate the volume that the “real structure” occupies. Measurements made on the basis of the structural image can be related to the surface (*A_A_*, *L_A_*, *P_A_*) or a line drawn on the image (*L_L_*, *P_L_*). The latter case is called the Cavaliere–Hacquert linear method. Among other applications, this method is used for the determination of the void fraction (*φ*) of the channel, which is defined as the volume fraction of the dispersed phase per unit volume of a two-phase mixture. In this case, the void fraction (*φ*) is equal to the ratio of the length of the section passing through the gas phase structure (*L_ki_*) in the two-dimensional image to the total length of the section outlined in the image. The section *L_k_* is a straight line that connects opposite walls of the channel in a two-dimensional image. It is influenced by the most characteristic flow elements inside the channel. The operation can be repeated several times to obtain the average value of the void fraction for a given image.

In investigations involving two-phase flow structures, different methods of image analysis are used to determine the geometric parameters of the flow structure, velocity of individual phases, vapor quality (*x*), and void fraction (*φ*) [33]. The void fraction (*φ*) is defined as the volume fraction of the gas phase with respect to the total volume. According to the rules of stereology, it can be determined by reducing one dimension of the three-dimensional image; that is, by reducing the volume of individual phases to the cross-sectional area. In this case, the static void fraction can be calculated as follows:

(7) $$\varphi =\frac{A_{v}}{A_{v}+A_{l}},$$

where *A_v_* is the area of the image occupied by the vapor phase and *A_l_* is the area of the image occupied by the liquid phase. If the process takes place in a circular minichannel, then the two-phase flow structures exhibit circular symmetry. Therefore, it can be assumed that the fraction of the gas phase in the total volume, determined for the two-dimensional image, is close to the real fraction [34]. By knowing the static void fraction, it is possible to determine the static vapor quality (for example, in the case of condensation or boiling process):

(8) $$x=\frac{\rho_{v}}{\left(\frac{\rho_{l}}{\varphi }-\rho_{l}+\rho_{v}\right)},$$

where *ρ_l_* and *ρ_v_* are the densities of liquid and vapor phases, respectively; and *φ* is the static void fraction. This formula can be used during flow without interphase slip [35,36].

**Figure 4:**
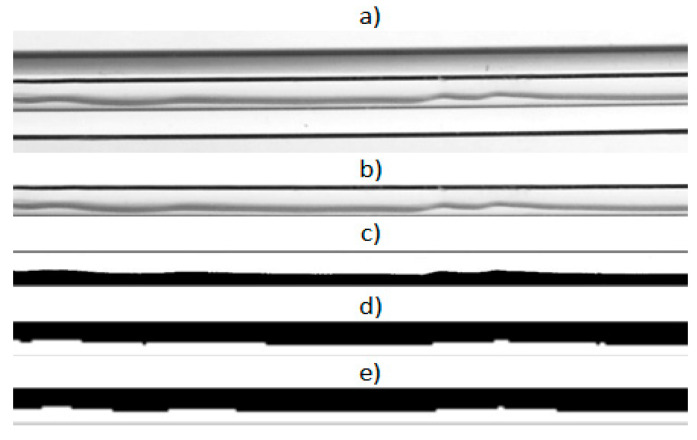
Graphical interpretation of the image processing steps before the analysis: (**a**) film frame; (**b**) image after cutting the interior of the minichannel; (**c**) binary image; (**d**) image after inverting the colors by the algorithm; (**e**) image after removing the noise by the algorithm [36,37].

This method was used for our investigations. In image analyses, frames were generated from the recorded images and processed. The image analysis used in this study was based on the algorithm presented by Michalska-Pożoga et al. [37], which was written in MATLAB (version, manufacturer, city, country) and modified by the author and adapted to determine the void fraction *φ*. The protocol followed for image processing and analysis is presented in Figure 4. Based on the results of this analysis, vapor quality x was determined using the dependencies defined by Equation (8).

To apply the above algorithm, it was necessary to capture the correct image of the tested flow structure to enable the algorithm to binarize the image. Figure 4 shows the individual phases of image processing using the described algorithm [36]. The first step was to cut an image of the inside of the minichannel from the test frame (that is, remove the background). This was necessary because the visualization studies were carried out inside the minichannel. The next step was the possible closing of the phase separation line and binarization of the image (Figure 4c). The black pixels now occupied the fields filled with the liquid phase, and the white pixels occupied the gas phase. Then, the algorithm inverted the image (Figure 4d) and reduced the so-called noise (Figure 4e). During inversion, the color of the pixels changed from white to black and vice versa, which meant that after inversion, the liquid phase was represented by white pixels and the gas phase by black pixels. Only such a prepared image could be analyzed using the MATLAB algorithm. The algorithm also allowed the author to align shapes and change colors, contrast, and brightness, as well as the filter. Based on the given dimensions of the image and the number of black and white pixels counted by the algorithm, it was possible to determine the area of the image occupied by individual phases. The ratio of the black pixel area (after image conversion and filtering by the algorithm) to the total area of the image described the void fraction *ϕ*. Based on this value, the vapor quality *x* was determined. A very similar method in the study of the flow structures during boiling was used by Płaczkowski et al. [38].

The accuracy of the determination of the void fraction and vapor quality using stereology technique depends on observed flow structures, the size of minichannel, the flow rate of the refrigerant, and the image recording rate. Accuracy varies from about 2 to 20%. The best accuracy of stereology methods is for plug and annular flows; the mist and transition structures, where froth is observed, have an accuracy of about 20%. The larger diameter of the minichannel gives a higher accuracy when determining the area occupied by individual phases. Accuracy also depends on the image quality.

## 4. Experimental Visualization Results

**Figure 5:**
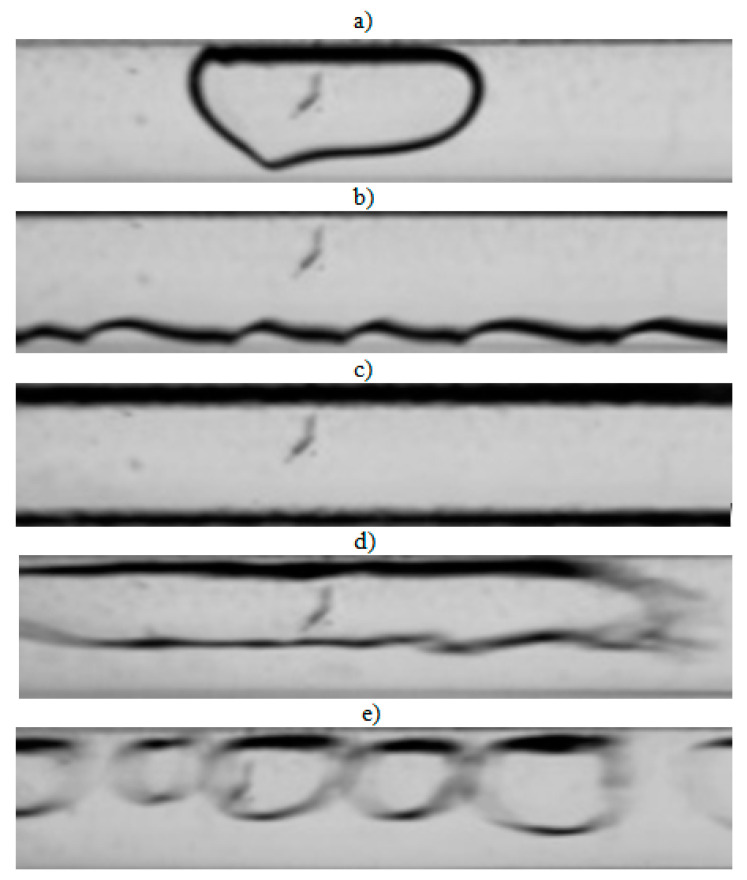
Experimental results of visualization research on HFE7000 refrigerant condensation in a minichannel with an internal diameter *d_h_* = 2.0 mm: (**a**) plug flow *G* = 88 kg/m^2^s, x = 0.002, *ϕ* = 0.27, *t_s_* = 29 °C; (**b**) annular-wavy flow *G* = 97 kg/m^2^s, x = 0.011, *ϕ* = 0.68, *t_s_* = 31 °C; (**c**) annular flow *G* = 97 kg/m^2^s, x = 0.011, *ϕ* = 0.68, *t_s_* = 31 °C; (**d**) slug flow *G* = 1168 kg/m^2^s, x = 0.006, *ϕ* = 0.55, *t_s_* = 31 °C; (**e**) bubbly flow *G* = 487 kg/m^2^s, x = 0.003, *ϕ* = 0.26, *t_s_* = 41 °C.

**Figure 6:**
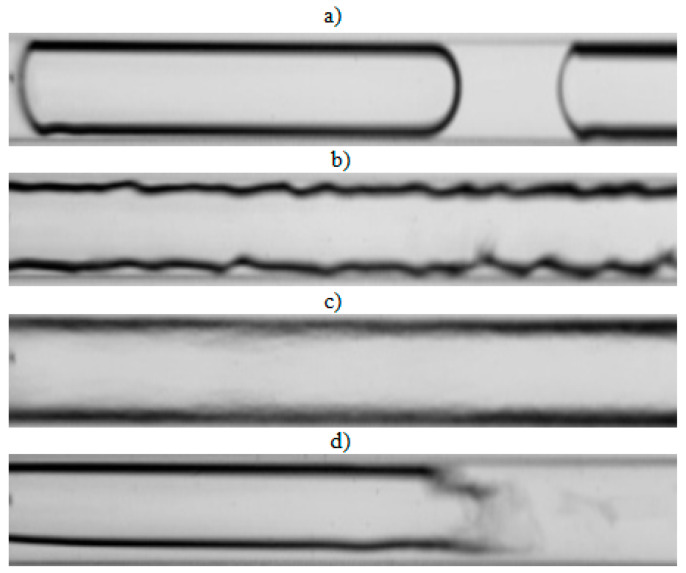
Experimental results of visualization research on HFE7000 refrigerant condensation in a minichannel with an internal diameter *d_h_* = 0.8 mm: (**a**) plug flow *G* = 197 kg/m^2^s, x = 0.004, *ϕ* = 0.44, *t_s_* = 31 °C; (**b**) annular-wavy flow *G* = 369 kg/m^2^s, x = 0.012, *ϕ* = 0.69, *t_s_* = 32.5 °C; (**c**) annular flow *G* = 491 kg/m^2^s, x = 0.032, *ϕ* = 0.82, *t_s_* = 41 °C; (**d**) slug flow *G* = 639 kg/m^2^s, x = 0.003, *ϕ* = 0.34, *t_s_* = 29 °C.

**Figure 7:**
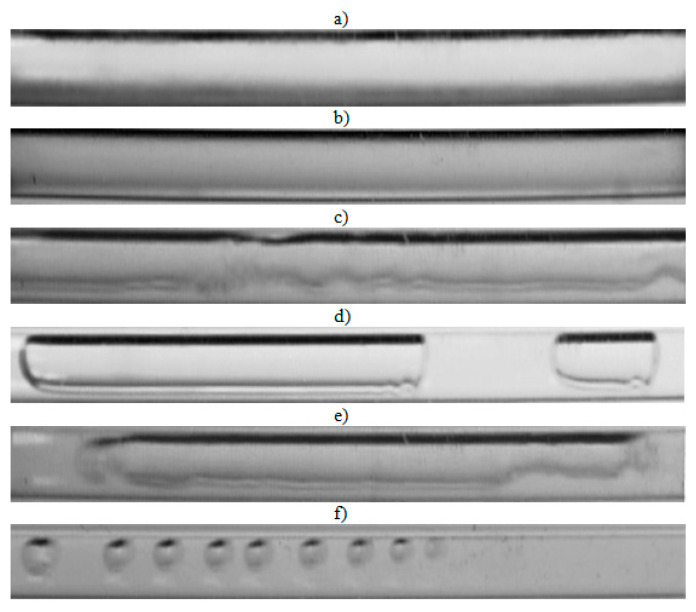
Experimental results of visualization research on HFE7100 refrigerant condensation in a minichannel with an internal diameter *d_h_* = 2.0 mm: (**a**) mist flow *G* = 265 kg/m^2^s, x = 0.82, *ϕ* = 0.97, *t_s_* = 59 °C; (**b**) annular flow *G* = 1291 kg/m^2^s, x = 0.5, *ϕ* = 0.87, *t_s_* = 58 °C; (**c**) annular flow *G* = 469 kg/m^2^s, x = 0.14, *ϕ* = 0.52, *t_s_* = 59 °C; (**d**) plug flow *G* = 309 kg/m^2^s, x = 0.1, *ϕ* = 0.43, *t_s_* = 59 °C; (**e**) slug flow *G* = 1415 kg/m^2^s, x = 0.18, *ϕ* = 0.59, *t_s_* = 59 °C; (**f**) bubbly flow *G* = 424 kg/m^2^s, x = 0.01, *ϕ* = 0.09, *t_s_* = 59 °C.

**Figure 8:**
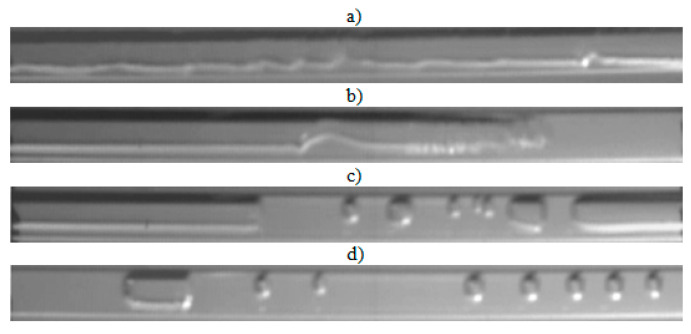
Experimental results of visualization research on HFE7100 refrigerant condensation in a minichannel with an internal diameter *d_h_* = 0.8 mm: (**a**) annular-wavy flow *G* = 3539 kg/m^2^s, x = 0.23, *ϕ* = 0.67, *t_s_* = 59 °C; (**b**) slug flow *G* = 1180 kg/m^2^s, x = 0.11, *ϕ* = 0.45, *t_s_* = 59 °C; (**c**) plug flow *G* = 1180 kg/m^2^s, x = 0.07, *ϕ* = 0.33, *t_s_* = 59 °C; (**d**) bubbly flow *G* = 1769 kg/m^2^s, x = 0.06, *ϕ* = 0.29, *t_s_* = 59 °C.

**Figure 9:**
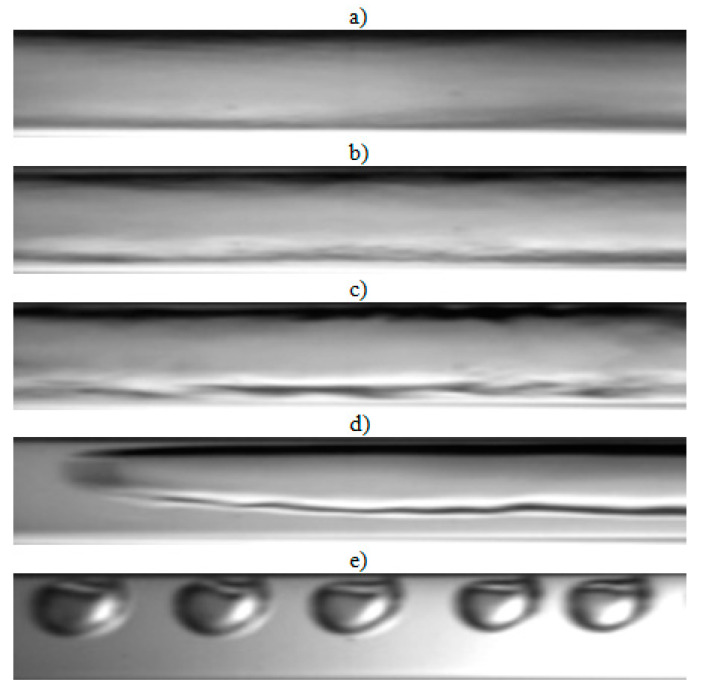
Experimental results of visualization research on Novec649 refrigerant condensation in a minichannel with an internal diameter *d_h_* = 1.2 mm: (**a**) mist flow *G* = 2187 kg/m^2^s, x = 0.042, *ϕ* = 0.82, *t_s_* = 61 °C; (**b**) annular flow *G* = 2015 kg/m^2^s, x = 0.029, *ϕ* = 0.75, *t_s_* = 54 °C; (**c**) annular-wavy flow *G* = 2236 kg/m^2^s, x = 0.015, *ϕ* = 0.63, *t_s_* = 51 °C; (**d**) slug flow *G* = 1966 kg/m^2^s, x = 0.008, *ϕ* = 0.48, *t_s_* = 59 °C; (**e**) bubbly flow *G* = 1032 kg/m^2^s, x = 0.003, *ϕ* = 0.27, *t_s_* = 51 °C.

**Figure 10:**
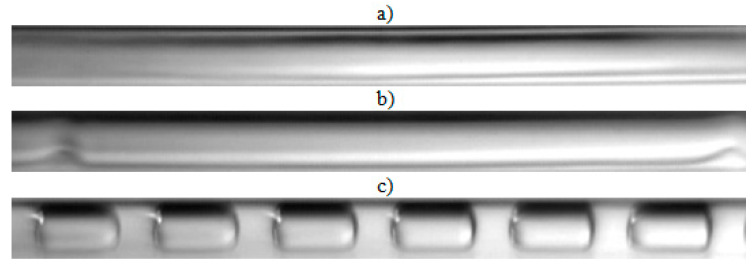
Experimental results of visualization research on Novec649 refrigerant condensation in a minichannel with an internal diameter *d_h_* = 0.5 mm: (**a**) annular flow *G* = 3114 kg/m^2^s, x = 0.056, *ϕ* = 0.77, *t_s_* = 72 °C; (**b**) annular-wavy flow *G* = 5379 kg/m^2^s, x = 0.023, *ϕ* = 0.57, *t_s_* = 71 °C; (**c**) plug flow *G* = 5379 kg/m^2^s, x = 0.019, *ϕ* = 0.53, *t_s_* = 71 °C.

The visualization studies of the two-phase flow structures formed during the condensation of HFE7000, HFE7100, and Novec649 refrigerants were performed on the test stand shown in Figure 1. This article presents the results characteristic of this study. Vapor quality and void fraction were determined using a picture analysis system described by Bohdal and Sikora [36]. Figure 5 and Figure 6 present the structures formed during the visualization of the condensation process of HFE700 refrigerant in minichannels with an internal diameter *d_h_* = 2.0 and 0.8 mm. For this refrigerant, structures such as mist, annular, annular-wavy, slug, plug, and bubble were produced. Figure 7 shows the results of the visualization of the condensation structures of HFE7100 refrigerant in a minichannel with a diameter *d_h_* = 2.0 mm. In this case, the following structures were observed: mist, annular, annular-wavy, slug, plug, and bubble. Exemplary flow structures, observed during the study of the condensation of the HFE7100 refrigerant in a minichannel with a diameter of 0.8 mm, are shown in Figure 8. Figure 9 and Figure 10 show pictures of flow structures observed during Novec649 refrigerant condensation in minichannels with internal diameter *d_h_* = 1.2 and 0.5 mm. Similar two-phase flow structures were observed with two diameters; however, during condensation in the channel with *d_h_* = 1.2 mm, no plug structure was observed. The descriptions given in Figure 5, Figure 6, Figure 7, Figure 8, Figure 9 and Figure 10 specify the parameters used during the two-phase condensation flow, which allowed for drawing conclusions regarding the occurrence of individual structures.

In Figure 5, Figure 6, Figure 7, Figure 8, Figure 9 and Figure 10, it can be seen that the type of flow structure depended on the flow parameters, such as mass flux density *G*, velocity of individual phases, vapor quality *x*, and void fraction *ϕ*. With the increase of the mass flux density, the range of occurrence of stratified structures decreased in favor of discontinuous ones. The plug and slug flows were included in the group of slug structures. They differed from each other in shape and thermal flow parameters. The slug flow was particularly observed for high mass flux densities *G*, the value of which depended on the type of refrigerant tested and vapor quality. It was related to the disturbances on the interface. Below *G* = 500 kg/m^2^s, there was practically no slug flow in the tested conditions, but above this value, plug flow disappeared in favor of slug flow. The plug flow occurred in the form of a regularly shaped slug, and was mainly observed in small-diameter channels, especially at *d_h_* ˂ 1 mm. Under these conditions, an increase in the influence of surface tension on the condensation process became apparent. At *G* > 3000 kg/m^2^s, no bubbly flow was observed.

## 5. Analysis of the Experimental Investigation Results

**Figure 11:**
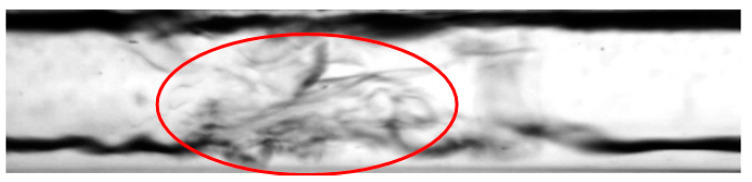
Frothy flow formation when a wave is refracted in a annular-wave structure during the condensation of the HFE7000 refrigerant in a minichannel with a diameter *d_h_* = 2.0 mm (*G* = 203 kg/m^2^s; x = 0.011; *ϕ* = 0.66; *t_s_* = 33 °C).

The flow regimes observed during the process of condensation in horizontal pipe minichannels can be classified into three main groups: dispersed flow, stratified flow, and intermittent flow. The dispersed structures include the mist and frothy substructures, while the stratified structures include the annular, annular wave, and wave substructures. Among the intermittent structures, the following substructures are distinguished: slug, plug, and bubble. Unfortunately, it is difficult to define the boundaries of transition between the structures due to the formation of the so-called transition or mixed structures. An example of such a phenomenon may be the phenomenon of “breaking of waves” in the annular wave structure, which occurs at high velocities of the vapor phase and causes the formation of froth (Figure 11).

**Figure 12:**
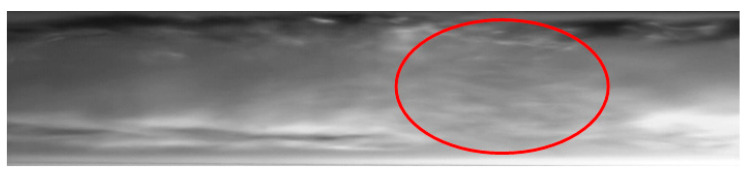
Froth formation in annular-wave flow at high speeds of both phases during condensation of Novec649 refrigerant in a minichannel with internal diameter *d_h_* = 2.0 mm (*G* = 1796 kg/m^2^s; x = 0.01; *ϕ* = 0.5; *t_s_* = 54 °C).

**Figure 13:**
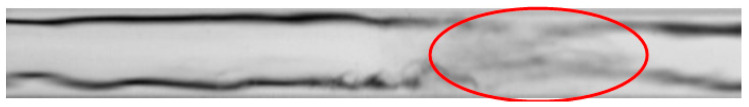
Formation of a slug structure during HFE7000 refrigerant condensation in a minichannel with an internal diameter *d_h_* = 0.8 mm (*G* = 418 kg/m^2^s; x = 0.008; *ϕ* = 0.62; *t_s_* = 29 °C).

**Figure 14:**
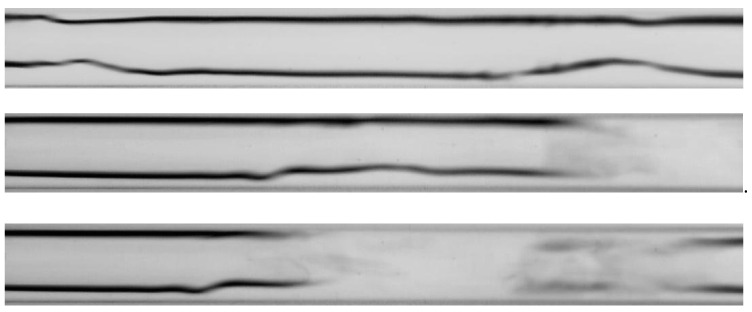
The successive phases of the transition from the annular-wave structure to the slug structure during the condensation of HFE7000 refrigerant in a minichannel with an internal diameter *d_h_* = 0.8 mm (*G* = 197 kg/m^2^s; x = 0.004; *ϕ* = 0.44; *t_s_* = 31 °C).

The froth substructure can also be formed in an annular or annular wave flow, at a very high velocity of the vapor and liquid phases, when the liquid phase is entrained as small droplets and mixes with the vapor phase. It results in the formation of so-called “sharp projections” in the flow. Such a case is presented in Figure 12. The consequence of the occurrence of high-amplitude waves and froth during the condensation process (with a decrease in the vapor quality and void fraction) is the transition to a slug structure. This phenomenon is shown in Figure 13. The transition of the flow structure from a stratified structure to an intermittent one is not always gradual, as shown in Figure 14. It was often possible to observe the simultaneous existence of two structures; for example, an annular structure and a mist structure. It is caused due to the formation of mist by the condensate droplets and their settling on the channel wall. A transitional structure occurs between the mist and the annular flow.

**Figure 15:**
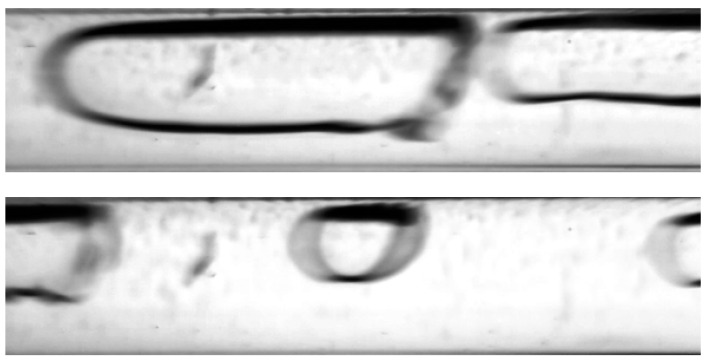
Example of the coexistence of a slug and bubble substructure during condensation of the HFE7000 refrigerant in a minichannel with an internal diameter of *d_h_* = 2.0 mm (*G* = 602 kg/m^2^s; x = 0.003; *ϕ* = 0.36; *t_s_* = 32 °C).

**Figure 16:**
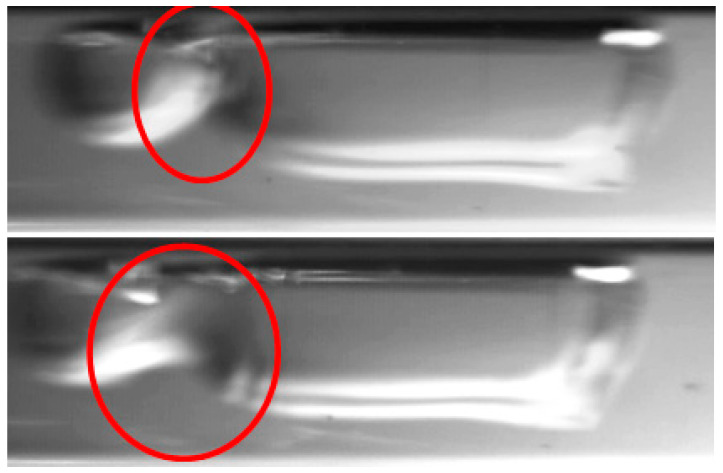
An example of the occurrence of vapor bubbles coalescing during the two-phase flow of Novec649 refrigerant in a minichannel with an internal diameter of *d_h_* = 2.0 mm (*G* = 2089 kg/m^2^s; x = 0.011; *ϕ* = 0.54; *t_s_* = 52 °C).

The other substructures that very often occur simultaneously are the slug and bubbly substructures or the plug and bubbly substructures. Figure 15 shows a record of the possible structures that can coexist during the condensation of HFE7000 refrigerant in a minichannel with a diameter *d_h_* = 2.0 mm. The reason for the coexistence of the slug and bubble substructures may be due to the phenomenon of coalescence, which is observed due to the merging of vapor bubbles in the flow, and thus resulting in the formation of slugs (Figure 15). Its occurrence is related to the differences in the velocity of the steam bubbles, which is further dependent on their size. In the case of an upward flow in a vertical channel, smaller bubbles have a higher velocity, which causes them to “catch up” with the supposedly larger ones and associate with them. In the case of downward flow in the vertical channel, larger bubbles “catch up” with the smaller ones [39]. Both of these cases were observed during two-phase flow in the horizontal channel. An example of vapor bubble coalescence is shown in Figure 16.

## 6. Maps of Two-Phase Flow during Condensation of Novec Refrigerants

**Figure 17:**
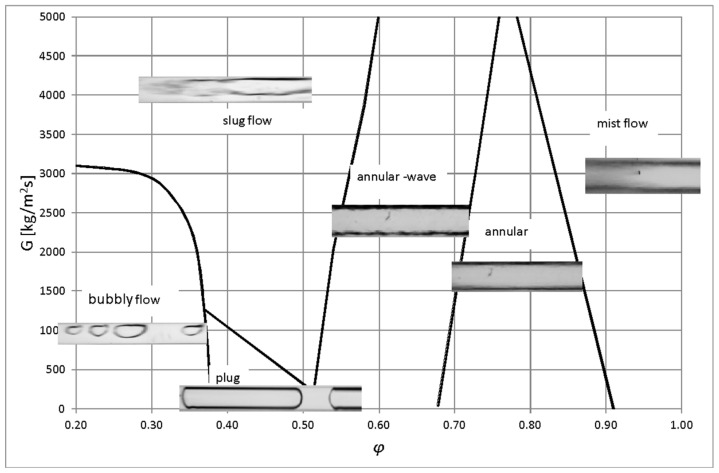
The proposition of a generalized flow structure map during HFE7000, HFE7100, and Novec649 refrigerant condensation in minichannels with an internal diameter of *d_h_* = 2.0 ÷ 0.5 mm.

**Figure 18:**
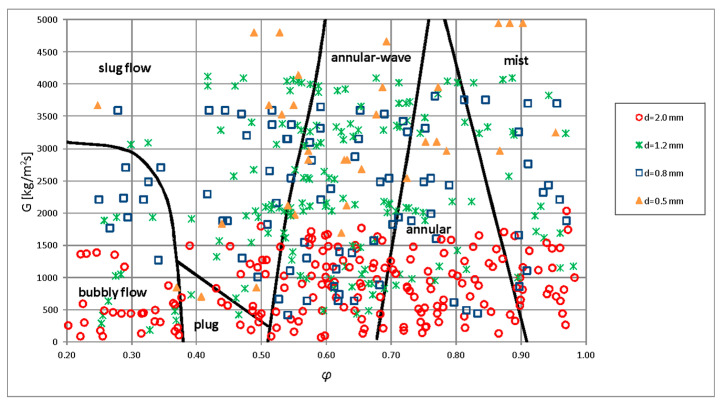
The proposition of a generalized flow structure map during HFE7000, HFE7100, and Novec649 refrigerant condensation in minichannels, taking into account the influence of the internal diameter.

Based on the results obtained after visualization studies on the condensation of HFE7000, HFE7100, and Novec649 refrigerants in a minichannel, the author constructed maps of flow structures. Owing to the fact that the HFE7100 refrigerant had a much higher vapor phase density compared to the HFE7000 and Novec649 refrigerants, significant difficulties were encountered during the construction of a common map of flow structures in *G*–x coordinates for the three tested refrigerants. In an attempt to generalize the results of experimental studies obtained for these three refrigerants, we proposed to present an experimental map of two-phase flow structures formed during condensation in minichannels with an internal diameter *d_h_* = 2.0–0.5 mm, in the coordinates *G*–*ϕ*. Figure 17 and Figure 18 present original maps of the flow structures created as a result of visualization investigations, and the construction of maps was supported by image analysis.

Figure 18 shows the influence of the channel diameter on the occurrence of individual flow structures. This was directly related to the mass flux density, which increased as the channel diameter decreased at a constant flow rate. Based on the presented flow structure map, it was concluded that the annular, plug, and bubbly substructures occurred mainly at lower mass flux density (*G*) values. As can be seen in Figure 17 and Figure 18, at the mass flux density *G* = 2500 kg/m^2^s, mist flow occurred with void fraction *φ* > 0.85, annular flow at 0.72 < *φ* < 0.85, annular wave flow at 0.55 < *φ* < 0.72, slug flow at 0.35 < *φ* < 0.55, and bubbly flow at *φ* < 0.35. In this range of mass flux densities, no occurrence of plug flow was observed. At high values of mass flux density (both phases at high velocities), the probability for the formation of mist, annular wave, and slug flow structures was widened, and the remaining observed substructures disappeared. With an increase in the velocity of the gas phase and the amount of condensate (with a decrease in the void fraction *φ*), the annular substructure transformed to the annular wave substructure. It was similar to intermittent structures. With the increase in the difference of velocity between the vapor and liquid phases, the plug substructure disappeared and underwent transition to the slug substructure. The plug substructure exhibited regular shapes, which resulted from the low value of the mass flux density (*G*), which in turn could be attributed to the low velocity of both phases and the relatively small difference in their velocities (slip).

**Figure 19:**
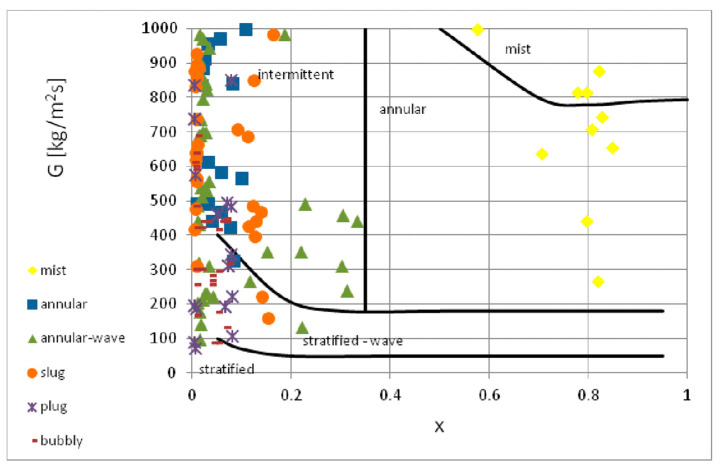
Comparison of the author’s experimental research results with the map of two-phase flow structures according to map of El Hajal et el. [40].

**Figure 20:**
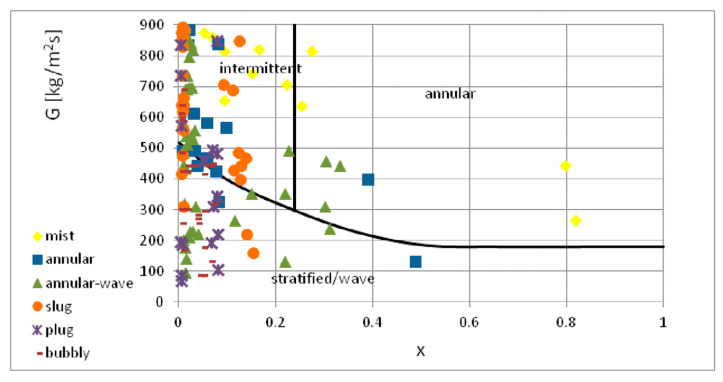
Comparison of the author’s experimental research results with the map of two-phase flow structures according to map of Olivier et al. [41].

**Figure 21:**
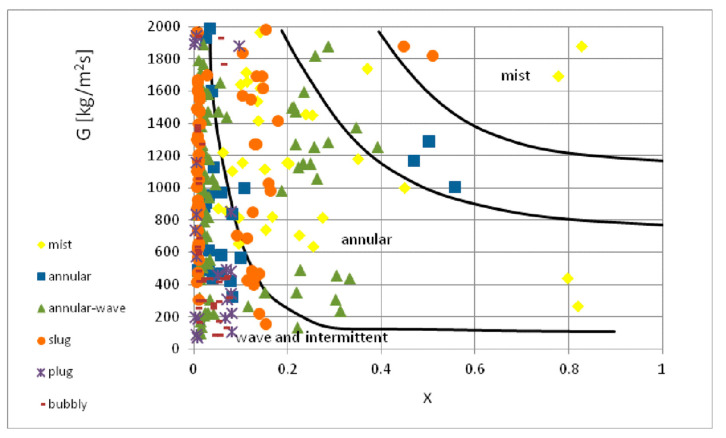
Comparison of the author’s experimental research results with the map of two-phase flow structures according to map of Soliman [42].

The results of the present study were compared with the flow structure maps proposed by other authors. It was observed that the maps presented in Figure 19, Figure 20 and Figure 21 had different ranges of mass flux density (*G*). Unfortunately, the ranges of the presented maps of flow structures only partially corresponded to the scope of the presented research. The map proposed by El Hajal [40] (Figure 19) has been verified for its possible application in two-phase flows in minichannels during the boiling process and in the presence of high-pressure refrigerants. In the case of condensation of the tested low-pressure refrigerants in the minichannels, the boundaries of the occurrence of individual structures were shifted towards lower values of the vapor quality.

Figure 20 depicts the map of two-phase flow structures proposed by Soliman [41]. It was created for the two-phase, nonadiabatic flow of R12 and R113 refrigerants in conventional channels. The lack of compliance also occurred when comparing this study’s experimental results with the map of two-phase flow structures developed by Olivier et al. [42] (Figure 21). They created the map for the condensation of high- and medium-pressure refrigerants in finned minichannels. It can be seen that this map does not take into account the occurrence of intermittent structures at low values of mass flux density (up to 300 kg/m^2^s). This was related to an increase in surface roughness due to microribbing. Such a phenomenon was not observed in the current study. Thus, the discrepancy in the results of experimental studies can be attributed to differences in both physicochemical properties of the tested factors and the dimensions of the channel.

## 7. Conclusions

Three main flow structures were observed during the condensation of HFE7000, HFE7100, and Novec649 refrigerants in pipe minichannels: dispersed, stratified, and intermittent. These structures were further divided into substructures as follows: dispersed structures included mist and frothy substructures; stratified structures included annular, annular wave, and wave substructures; and intermittent structures included slug, plug, and bubble substructures. During the study, the frothy and wave substructures were the least frequently observed, while the most common were annular, annular wave, and slug substructures. During the visualization investigations, phenomena such as transition between structures, bubble coalescence, or the coexistence of two flow structures at the same time were observed. The following conclusions can be drawn from the presented research results:

(1) Based on the research, it was found that at the mass flux density *G* = 2500 kg/m^2^s, mist flow occurred with void fraction *ϕ* > 0.85, annular flow at 0.72 < *ϕ* < 0.85, annular wave flow at 0.55 < *ϕ* < 0.72, slug flow at 0.35 < *ϕ* < 0.55, and bubbly flow at *ϕ* < 0.35.
(2) During visualization investigations of condensation, flow structures were observed, and at high values of mass flux density (high velocities of both phases), the range of mist, annular wave, and slug flow occurrence was widened, and the remaining observed substructures disappeared. With an increase in the velocity of the gas phase and the amount of condensate (with a decrease in the void fraction *φ*), the annular substructure transformed into the annular wave substructure, which was similar to the intermittent structures. With the increase of the difference in velocity of the vapor and liquid phases, the plug substructure disappeared and underwent a transition to the slug substructure. The plug substructure exhibited regular shapes, which resulted from the low value of the mass flux density (*G*), which in turn was reflected by the low velocity of both phases and the relatively small difference in their velocities.
(3) The influence of the minichannel diameter on the formation of flow structures was visible. In minichannels with an internal diameter smaller than 1 mm, the influence of surface tension was clearly visible, which resulted in a wider range of plug and bubbly flow. No slug flow was observed in such small channels.
(4) The results of the author’s research were compared with the flow structure maps presented by Soliman [41] and Olivier et al. [42]. This comparison did not give satisfactory results due to discrepancies in the scopes of the studies and the applicability of the above studies. Unfortunately, so far no other flow structure maps have been developed for the condensation process of low-pressure media in minichannels.
(5) The novelty of the presented research is the scope. So far, there are no results of studies on flow structures during the condensation of Novec refrigerants in minichannels. There are no flow structure maps for the condensation process of these refrigerants, especially in minichannels or for high mass flux densities.
(6) It is important to combine visualization investigations of flow structures with thermal studies of the condensation process to determine their influence on the intensity of heat transfer. Research in this aspect is being conducted by the author.

## Data Availability

The data presented in this study are available on request from the corresponding author.

## Nomenclature

A	cross section (m^2^)
d	diameter (m)
*G*	mass flux density (kg/m^2^s)
g	gravity forces (m/s^2^)
j	superficial velocities (m/s)
L	length (m)
m	mass flux (kg/s)
P	point
t	temperature (°C)
T	temperature (K)
x	vapor quality (-)
ρ	density (kg/m^3^)
*ϕ*	void fraction (-)
σ	surface tension (N/m)
*Bo*	Bond number
*Fr*	Freund number
*We*	Weber number
χ_tt_	Lockhart–Martinelli parameter
Subscripts	
cr	critical
h	hydraulic
l	liquid
s	saturation
v	vapor
